# Assessment of synergistic effects of ultrasonication, ozonation and their combination with aloe vera active edible coating containing *spirulina* and turmeric extracts on preservation of fresh-cut apples

**DOI:** 10.1016/j.ultsonch.2026.107913

**Published:** 2026-06-04

**Authors:** Samran Khalid, Kashmala Chaudhary, Ahmed Fathy Ghazal, Abderrahmane Aït-Kaddour, Marwa Ezz El-Din Ibrahim, Rana Muhammad Aadil

**Affiliations:** a National Institute of Food Science and Technology, University of Agriculture, Faisalabad, 38000, Pakistan; b Food Science Program, Division of Food, Nutrition and Exercise Sciences, University of Missouri, Columbia, MO 65211, United States; c School of Food Science and Engineering, South China University of Technology, Guangzhou 510641, China; d Agricultural Engineering Department, Faculty of Agriculture, Suez Canal University, Ismailia 41522, Egypt; e Université Clermont Auvergne, INRAE, VetAgro Sup, UMRF, 63370 Lempdes, France; f Dept. Food Science and Technology, IPB University, IPB Dramaga Campus, Bogor 16680, West Java, Indonesia; g Department of Food and Nutrition Sciences, College of Agricultural and Food Sciences, King Faisal University, Al-Ahsa 31982, Saudi Arabia

**Keywords:** Aloevera gel, Active edible coating, Non-thermal preservation, Synergistic effect, Quality retention, Shelf-life extension

## Abstract

Fresh-cut apples (FCA) are widely consumed due to their convenience and high nutritional value; however, their quality declines rapidly during storage due to enzymatic browning, microbial proliferation, oxidative reactions, and moisture loss. While edible coatings and non-thermal processing approaches have been studied independently for postharvest preservation of fresh-cut produce, their combined use along with natural bioactive compounds has received limited attention. Therefore, this study evaluated the effectiveness of a novel aloe vera gel-based active edible coating enriched with *Spirulina platensis* and turmeric extracts, applied alone (CT) or in combination with ultrasonication (US), ozonation (OZ), and their combined treatment (US + OZ) for preserving FCA at 4 °C for 42 days. Multiple quality parameters including weight loss, firmness, color attributes, physicochemical properties, antioxidant compounds, oxidative stress markers, enzymatic activity, microbial load, and overall acceptability were assessed throughout the storage. The coating treatment alone (CT) showed the most effective preservation, limiting weight loss to 4.58% after 42 days and maintaining firmness at 12.75 N, while the untreated control deteriorated rapidly with 17.41% weight loss and severe texture loss by day 14. Microbial growth remained minimal in CT (2.91 log CFU/ g total plate count; <2.5 log CFU/g yeast and mold). CT also preserved bioactive compounds with total phenolics (4.41 mg GAE/g), flavonoids (1.43 mg QE/g), and antioxidant activity (44.37% DPPH). The combined US + OZ-assisted coating treatment also exhibited strong preservation performance, particularly in maintaining firmness, reducing oxidative stress accumulation, and suppressing enzymatic browning-related activities. The improved preservation efficacy was associated with enhanced barrier properties of the coating matrix, reduced oxidative degradation, inhibition of browning-associated enzymes, and improved microbial control induced by the integrated non-thermal treatments. Overall, the aloe vera-based active coating, either alone or combined with mild US + OZ treatment, effectively maintained quality attributes and extended the storage stability of FCA up to 42 days compared with the rapid deterioration observed in untreated samples within 14 days. These findings highlight the potential of integrating natural bioactive coatings with non-thermal technologies as a sustainable preservation strategy for fresh-cut fruit applications.

## Graphical abstract

**Figure:**
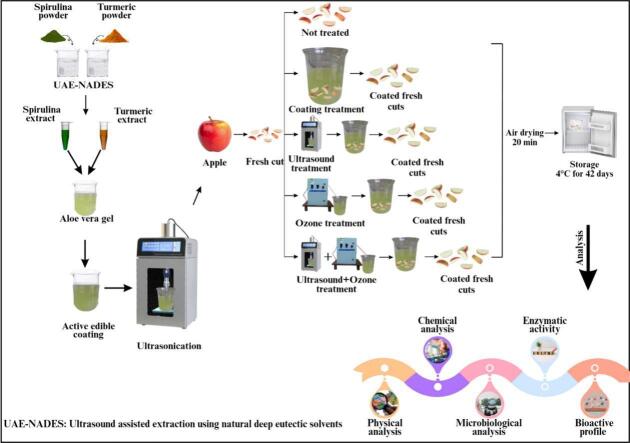

## 1 Introduction

Fresh-cut products defined as horticultural commodities that undergo washing, trimming, peeling, and cutting into ready-to-eat portions while remaining minimally processed offer consumers both convenience and nutritional benefits [1], [2], [3]. They are vital in modern food systems as in today's fast-paced world, where convenience and nutrition are equally prioritized, fresh-cut products offer time-saving solutions while maintaining the health benefits of whole produce [4], [5], [6]. Among these, fresh-cut apples (FCA) are widely consumed worldwide and are recognized as a nutrient-rich option suitable for daily intake [3], [7]. However, like all fresh-cut produce, they are highly susceptible to rapid quality deterioration through moisture loss, texture softening, aroma depletion, nutrient degradation, enzymatic browning, microbial spoilage, and overall acceptability decline [2], [6], [7], [8], [9], [10]. Compared to whole fruits, FCA exhibit a higher susceptibility to spoilage as a result of multiple factors [3]. Current preservation methods present several limitations and are not so effective in increasing shelf life for a longer time. Synthetic preservatives may leave harmful residues or compromise flavor profiles, controlled atmosphere storage requires substantial energy inputs and expensive infrastructure, while conventional cold storage can induce chilling injuries, elevate operational costs, and gradually diminish product quality [8], [11], [12].

These limitations underscore the urgent need for effective innovative preservation approaches, including natural preservatives, advanced packaging systems, and non-thermal technologies [3]. While traditional packaging served basic functions of containment, protection and labeling, modern innovations have transformed its role in food preservation [1], [12]. Among these developments, edible coatings are increasingly recognized as efficient preservation strategies that reduce postharvest deterioration and enhance the storage stability of highly perishable products such as FCA [1], [3], [5], [7]. Edible coatings formulated from natural biopolymers generate semi-permeable barriers on food surfaces that effectively regulate factors associated with spoilage. These innovative materials can simultaneously preserve fresh-produce quality, ensure microbial safety, maintain bioactivity, and extend shelf-life [2], [3], [9], [13]. As sustainable packaging alternatives, these can offer distinct advantages over conventional plastics being biodegradable, cost-efficient at industrial scales, and chemically inert for food contact applications [14].

Aloe vera gel is a natural, biodegradable biopolymer with superior film-forming capacity, transparency, and inherent antimicrobial and antioxidant properties, making it a suitable carrier matrix for fresh-cut fruit preservation [15], [16]. However, its preservation efficacy alone is sometimes insufficient for extended storage stability. Considering the intrinsic limitations of biopolymers in food preservation, recent research efforts have increasingly focused on improving their functional performance through advanced modification strategies [17]. The integration of functional active agents into edible coatings represents a notable advancement in the evolution of active biodegradable packaging systems [7], [18]. These active edible coatings demonstrate improved functional properties, including enhanced antimicrobial and antioxidant activity, along with superior barrier performance against oxygen and moisture transmission [9], [17], [19], [20]. Active packaging systems operate by dynamically interacting with food products or their surrounding environment to regulate critical factors affecting shelf-life, such as atmospheric composition and microbial proliferation. In alignment with global sustainability goals, contemporary formulations increasingly utilize natural active agents derived from plant extracts, animal byproducts, and algal sources, which not only boost preservation efficacy but also maintain environmental compatibility [17], [21]. To reinforce its functionality, *S. platensis* and turmeric extracts were incorporated due to their rich bioactive profiles and complementary protective mechanisms. *S. platensis* provides a rich profile of bioactive constituents, including high-quality proteins, essential amino acids, polyunsaturated fatty acids, and antioxidant compounds such as phycocyanin, β-carotene, and phenolic*s* [22]. These components effectively scavenge free radicals, inhibit lipid oxidation, and exert broad-spectrum antimicrobial activity attributes that help delay enzymatic browning and microbial deterioration in fresh-cut produce [23]. Similarly, turmeric contains curcuminoids (primarily curcumin), essential oils, polysaccharides, phenolics, and flavonoids, contribute to enhance functional bioactivity. The incorporation of turmeric into edible coating systems improves oxidative resistance while strengthening antimicrobial barrier properties [24], [25]. Therefore, the synergistic incorporation of *S. platensis* and turmeric into the aloe vera gel matrix results in a multifunctional active edible coating capable of maintaining the nutritional, sensory, and microbiological quality of FCA during storage making it an innovative, safe, and sustainable alternative for postharvest preservation.

Furthermore, non-thermal technologies have gained increasing attention as efficient sustainable strategies for maintaining the quality of fresh-cut produce [8]. Among these approaches, ultrasound or ultrasonication (US) and ozone treatment or ozonation (OZ) methods have shown particular effectiveness for fresh-cut fruits; US operates through high-frequency sound waves (20–100 kHz) that induce cavitation, whereas OZ utilizes ozone (O_3_) as a strong oxidizing agent for microbial and enzymatic control. [6], [26], [27], [28]. The research has shown that compared with other non-thermal techniques, US and OZ are relatively low-cost, environmentally friendly, and easily adaptable for industrial-scale operations, making their combined use highly effective in prolonging the storage duration of fresh-cut fruits. In contrast, HPP and PEF require expensive equipment and are less effective for irregularly shaped or fragile fresh-cut fruits, PL may cause surface discoloration and quality degradation due to heating, CP often leads to tissue dehydration or localized oxidative damage, and IR can negatively affect sensory attributes and consumer acceptance [8], [29], [30]. These complementary advantages support the synergistic application of US and OZ for maintaining product quality and extending the shelf life. The combination of active edible coatings (incorporated with natural preservatives) and eco-friendly non-thermal technologies holds significant promise for fresh-cut produce preservation [3], [10], [11], [31], [32].

This study aimed to elevate FCA preservation by delaying overall deterioration through applying a novel active aloe vera gel edible coating, enriched with *S. platensis* and turmeric extracts, alone or in combination with sustainable non-thermal technologies (US, OZ, and US + OZ). It was hypothesized that the integration of active edible coating with non-thermal preservation technologies would enhance microbial inactivation, suppress enzymatic browning and oxidative deterioration, and consequently improve the physicochemical stability and storage quality of FCA during refrigerated storage. Furthermore, the combined treatments were expected to provide superior preservation efficacy through complementary barrier, antioxidant, and non-thermal effects compared with individual treatments alone. This study addresses a critical gap in FCA preservation by utilizing a sustainable method that maintains quality, ensures safety, retains bioactivity, and extends shelf life. While previous studies have explored edible coatings, active coatings, non-thermal technologies, and their combinations as hurdle technologies for FCA preservation, the reported shelf life under refrigerated storage typically does not exceed about 23 days, depending on treatment conditions and packaging strategies [1], [3], [9], [31], [44], [49], [50]. Therefore, this work contributes to the development of next-generation natural preservation systems aimed at reducing postharvest losses and improving the sustainability of fresh-cut produce processing. Moreover, the recent advances in functional material engineering, adsorption-based systems, and oxidative-regulation strategies in related scientific fields further emphasize the importance of designing multifunctional materials with enhanced barrier, stability, and protective properties for preservation-related applications [53], [54].

## 2 Materials and methodology

### 2.1 Materials

Analytical-grade chemicals and reagents employed in this work were sourced from Sigma-Aldrich (St. Louis, MO, USA). Aloe vera leaves were collected from a local garden, while turmeric powder was obtained from a local commercial source. *S. platensis* powder was sourced from Daraz.pk, Pakistan. Gala apples (*Malus domestica* Borkh., cv. Gala) were harvested at the commercial maturity stage from an orchard in the Swat Valley, Pakistan. Fruit maturity was confirmed based on firmness (12.50–14.50 N) and total soluble solids (TSS) content (14.0–14.5°Brix). Apples of consistent size, shape, and color, free from visible defects or contamination, were selected. The samples were then randomly assigned to experimental groups and transported to the laboratory under refrigerated conditions for further analysis.

### 2.2 Preparation of extracts by ultrasound assisted extraction (UAE)

UAE has gained considerable attention as a sustainable and efficient extraction technology because acoustic cavitation enhances solvent penetration, disrupts cellular structures, improves mass transfer, reduces extraction time and solvent consumption, and facilitates the recovery of bioactive compounds while preserving their functional properties [57]. So, we obtained the bioactive extracts from *S. platensis* and turmeric using UAE method in combination with natural deep eutectic solvents (NADES), based on our previously optimized protocol given in details in our previous studies [33], [34]. Dried powders were dispersed in NADES and preconditioned before UAE. After treatment, the mixtures were centrifuged, and the resulting supernatants were used for subsequent analyses.

### 2.3 Preparation of active edible coating solutions

Aloe vera leaves were first rinsed with chlorinated water and immersed for 15 min to eliminate aloin, a bitter yellow–brown compound present in the latex layer. After cleaning, the outer rind and spines were carefully removed, and the inner gel was collected. The extracted gel was subsequently subjected to homogenization using an Ultra-Turrax T25 homogenizer (IKA Labortechnik, Breisgau, Germany) operated at 10,000 rpm for 10 min to achieve a consistent and uniform dispersion. The homogenate was subsequently filtered through cheesecloth to remove fibrous residues and obtain a smooth gel fraction. To minimize enzymatic browning and stabilize the pH of the gel matrix, ascorbic acid (1%, w/v) and citric acid (1%, w/v) were incorporated. The edible coating formulation was prepared by combining 50% (v/v) aloe vera gel with 2% (w/v) glycerol, which served as a plasticizing agent, and 1% (w/v) eggshell powder with an average particle size of approximately 100 μm. The eggshell powder, mainly composed of calcium carbonate, was included as a mineral filler to reinforce the coating structure. Previous studies have demonstrated that eggshell-derived calcium carbonate can improve the mechanical strength, compactness, and barrier properties of biodegradable films by reducing polymer chain mobility and limiting the diffusion of water vapor and oxygen through the polymer network. Eggshell fillers have also been reported to reduce water vapor permeability and oxygen permeability in starch-, protein-, and polysaccharide-based packaging systems. In the present study, although direct permeability characterization was not performed, the incorporation of eggshell powder was indirectly associated with improved storage stability, including reduced weight loss, delayed firmness deterioration, and suppression of oxidative browning changes during refrigerated storage. The eggshell powder was obtained from food-grade hen eggs, thoroughly washed, boiled for 10 min for microbial decontamination, dried at 60 °C, finely ground, and sieved to obtain particles of approximately 100 μm. Previous literature has widely reported eggshell-derived calcium carbonate as a safe, biodegradable, and food-grade additive for food and packaging applications [51]. Based on our previously optimized internal experiments, which assessed various active edible coatings of aloe vera gel added with different concentrations of *S. platensis* and turmeric extracts alone or in combination, with or without US for whole apple preservation, the most effective formulation was identified as aloe vera gel containing *S. platensis* (5.0%) and turmeric (5.0%) extracts treated with US (power 200 W, frequency 20 kHz, time 10 min and temperature 25 °C) to improve its effectiveness. This formulation was selected for the present study.

### 2.4 Non-thermal treatment and coating application on fresh-cut apples (FCA)

Apples were cleaned and sanitized using a 0.1 g/L sodium hypochlorite solution, rinsed with a clean cloth, and dried before further processing. All knives, cutting boards, and utensils were sanitized with 700 mL/L ethanol and thoroughly dried to ensure hygienic handling. The apples were then cut into uniform slices using a sterilized knife for the treatments. A control group (NT) consisted of FCA without any treatment, while the experimental groups underwent different preservation treatments. For the coating-only treatment (CT), FCA were directly immersed in the previously prepared edible coating solution and kept submerged for 10 min to ensure uniform coating coverage. In the US-assisted treatment (UC), apple samples were subjected to probe-type US under pulsed conditions (10 s on / 5 s off) at a frequency of 25 kHz and an output power of 300 W for a duration of 5 min. To control temperature rise during processing, the treatment vessel was maintained in an ice bath, keeping the system at approximately 20 °C and minimizing thermal effects. Immediately after sonication, the samples were transferred into the coating solution and held for 10 min to facilitate the formation of an active layer on the fruit surface.

**Fig. 1:**
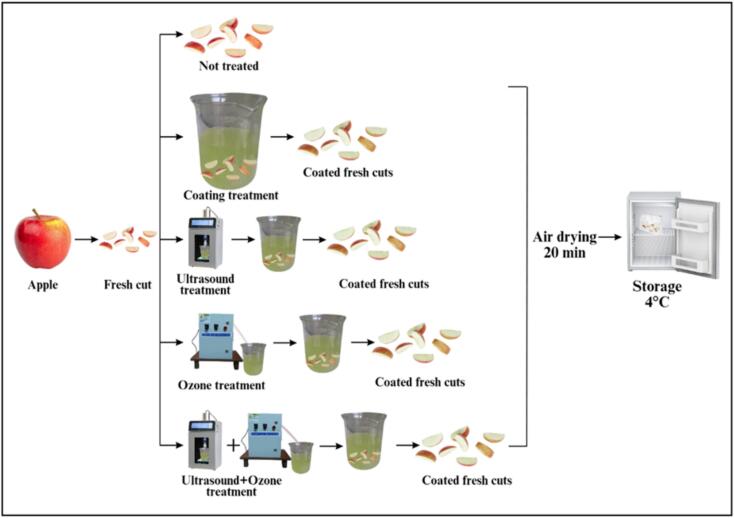
Schematic representation of the fresh-cut apples treatment process. NT (No treatment), CT (Coating only), UC (Ultrasonication followed by coating), OC (Ozonation followed by coating), and UOC (Combined ultrasonication and ozonation followed by coating).

In the third treatment (OC), apples were treated with aqueous ozone produced using an ozone generator (Ozonia® TOGC, Saint-Maurice, Switzerland). Ozone gas was bubbled into 5 L of deionized water at a generation rate of 800 mg h^−1^ for 30 min to obtain a stable dissolved ozone concentration of 1.5 mg L^−1^. The dissolved ozone concentration was continuously monitored using a Q45H/64 Dissolved Ozone Transmitter (Analytical Technology Inc., Collegeville, PA, USA). The ozone solution was prepared at approximately 20 ± 2 °C under continuous bubbling to facilitate mass transfer and maintain homogeneous ozone distribution. FCA slices were dipped in the freshly prepared aqueous ozone solution for 5 min, ensuring treatment occurred immediately after the target ozone concentration was reached to minimize ozone decay. Gentle mixing of the solution was maintained during treatment to ensure uniform exposure of apple slices. The residual ozone concentration was periodically monitored during treatment, and the short treatment duration minimized the loss of dissolved ozone due to its natural decomposition in water. After OZ, apples were dipped in the coating solution for 10 min. In the fourth treatment (UOC), apples were treated with both US and OZ, each applied for 2.5 min under the parameters mentioned above, before being dipped in the coating solution for 10 min. Following treatment, all apples were air-dried for 20 min before being stored at 4 °C for 42 days (Fig. 1). Samples were placed in sterile polypropylene trays and maintained under identical refrigerated conditions in a humidity-controlled produce storage refrigerator (∼90–95% RH) throughout the experimental period. Relative humidity was monitored through the refrigerator’s integrated humidity control/display system. Apples that exhibited spoilage were removed from the study to ensure accurate assessments. The US and OZ treatment parameters were selected based on preliminary trials (data not shown), ensuring that the chosen conditions maximized preservation efficacy while minimizing potential structural damage. Higher power, frequency, and flow rate settings were excluded due to their adverse effects on apple quality.

### 2.5 Analysis of fresh-cut apples (FCA) during storage

#### 2.5.1 Weight loss and firmness

Weight loss was evaluated by recording the mass of FCA slices during storage. The initial weight of slices from each treatment was measured using a digital balance (±0.01 g), and the same samples were reweighed at weekly intervals. The firmness of slices was evaluated using a texture analyzer (HDPlus, Stable Micro Systems, UK) equipped with a 5 mm probe.

#### 2.5.2 Color parameters, browning index (BI) and decay incidence

The color of the FCA slices was measured using a colorimeter, with results recorded according to the CIE (L*, a*, b*) color scale to assess changes in lightness, redness/greenness, and yellowness/blueness over time. These color parameters were used to calculate the BI using **Equations** (1), (2). The degree of decay was visually assessed by counting the number of decayed FCA in each replication and expressing it as a (%) of the total number of fruits, as shown in **Equation** (3).

(1) $$BI\left(\%\right)=\frac{\left(100\times (x-0.31\right))}{0.172}$$

Where x is calculated by Eq (2).

(2) $$x=\frac{\left(a*+1.75L*\right)}{\left(5.645L*+a*-3.012b*\right)}$$

(3) $$Decay\;Incidence\left(\%\right)=\frac{Number\;of\;decayed\;apples\;}{\mathrm{Total}\;\mathrm{number}\;\mathrm{of}\;\mathrm{apples}}\times 100$$

#### 2.5.3 Ascorbic acid

Ascorbic acid content was quantified using the 2,6-dichlorophenolindophenol (DCPIP) titration method according to Chettri et al. [35]. Apple juice (5 mL) was extracted in 0.5% oxalic acid to stabilize vitamin C, followed by filtration. The resulting extract was titrated against standardized DCPIP solution until the endpoint was reached.

#### 2.5.4 pH, total soluble solids (TSS), and titratable acidity (TA)

The sample pH was determined using a digital pH meter, while TSS were determined with a digital refractometer to estimate sugar content. TA was determined by diluting 10 mL of juice to 100 mL with distilled water, followed by titration with 0.1 N NaOH until the pH reached 8.0.

#### 2.5.5 Total phenolic content (TPC), total flavonoid content (TFC), and antioxidant activity

TPC, and TFC were quantified following a modified approach based on Ebrahimi & Rastegar. [36]. Apple pulp was extracted with 80% methanol, centrifuged, and the supernatant was used for analysis. TPC was determined using the Folin–Ciocalteu colorimetric assay using gallic acid as the standard reference compound. TFC was quantified using the aluminum chloride colorimetric method with quercetin used as the reference standard. Antioxidant activity was evaluated using the DPPH radical scavenging assay following the method of Gugan M et al. [52] with some modifications. Ascorbic acid was used as the positive control/reference antioxidant. Absorbance was measured at 750, 415, and 517 nm using a UV–Vis spectrophotometer (Shimadzu UV-2600, Japan).

#### 2.5.6 Hydrogen peroxide (H_2_O_2_) content, O_2_^−^ production rate and malondialdehyde (MDA) content

ROS levels in FCA samples were evaluated by determining H_2_O_2_, O_2_^−^ generation rate, MDA content using a modified spectrophotometric methods. H_2_O_2_ and MDA were determined following a modified procedure based on based on Saleem et al. [20], while O_2_^−^ generation was assessed using the hydroxylamine-based method. For H_2_O_2_ analysis, samples were extracted with 0.2% TCA, centrifuged, and the supernatant was reacted with PBS and potassium iodide prior to absorbance measurement at 390 nm. The O_2_^−^ generation rate was assessed by extracting samples in PBS, followed by reaction with hydroxylamine hydrochloride and subsequent color development using p-aminobenzene sulfonamide and α-naphthylamine, with absorbance recorded at 530 nm. Lipid peroxidation was determined as MDA using the TBA assay after extraction and heating, and absorbance was measured at 450, 532, and 600 nm.

#### 2.5.7 Enzymatic activities

The activities of antioxidant and browning-related enzymes were determined spectrophotometrically. Superoxide dismutase (SOD) activity was measured based on its ability to inhibit nitro-blue tetrazolium reduction under light exposure, with absorbance recorded at 560 nm. Peroxidase (POD) activity was evaluated using the guaiacol–H_2_O_2_ system by monitoring the increase in absorbance at 470 nm. Lipoxygenase (LOX) activity was determined using linoleic acid as a substrate, with absorbance measured at 234 nm. Polyphenol oxidase (PPO) activity was assessed using catechol as a substrate, and the formation of reaction products was monitored at 525 nm. Enzyme extracts were prepared in appropriate phosphate buffer systems and clarified by centrifugation prior to analysis. Enzymatic activities were expressed as units per gram fresh weight of tissue (U g^−1^ FW).

#### 2.5.8 Microbial analysis

Microbial populations were assessed based on a modified protocol adapted from Mehra et al. [37] with slight modifications. For sample preparation, 10 g of apple slices were aseptically homogenized in 90 mL of sterile 0.1% peptone water to obtain an initial dilution of 10^−1^, followed by serial decimal dilutions up to 10^−^⁶. For total plate count, 0.1 mL of appropriate dilutions was spread onto plate count agar and incubated at 37 °C for 24–48 h to enumerate aerobic mesophilic bacteria. Yeast and mold counts were determined by plating 0.1 mL of diluted samples onto acidified potato dextrose agar (PDA) to suppress bacterial growth. Yeast plates were incubated at 25–28 °C for 3–5 days, whereas mold plates were incubated at the same temperature for 5–7 days until visible colonies developed. Based on the dilution scheme and plating volume, the detection limit was approximately 1.0 log CFU g^−1^. Samples showing no detectable microbial colonies were recorded as < 1.0 log CFU g^−1^, and for statistical analysis these values were conservatively treated as 1.0 log CFU g^−1^ (the detection limit value) to avoid underestimation of microbial presence near the lower quantification threshold. All analyses were performed in triplicate for each treatment and sampling interval.

#### 2.5.9 Overall visual acceptability

The overall visual acceptability of FCA slices was evaluated using a 4-point visual quality scoring system, following the method described by Ebrahimi & Rastegar. [36]. Visual evaluations were performed independently by three trained laboratory researchers under standardized laboratory lighting conditions. Prior to evaluation, the panelists were familiarized with the scoring criteria and representative quality attributes associated with browning intensity, dehydration, firmness loss, visible microbial spoilage, and overall surface appearance. The samples were examined directly within their storage containers without tasting or extensive handling in order to preserve sample integrity and minimize contamination risk during long-term microbiological storage studies. The scoring system was defined as follows: 4 = excellent quality (fresh appearance, firm texture, no visible browning, dehydration, or microbial growth); 3 = good quality (slight browning, minor softening, and minimal surface dehydration without visible microbial growth); 2 = not saleable but still edible (moderate browning, noticeable softening, surface dehydration, and early signs of deterioration); and 1 = poor quality (severe browning, mushy texture, extensive dehydration, strong off-odor, and visible microbial spoilage). Final scores were calculated as the average of the three independent evaluations conducted at each sampling interval. Although the visual scoring system provided practical assessment of overall appearance deterioration and commercial acceptability trends during storage, the authors acknowledge that visual evaluation remains partially subjective and was not designed to definitively distinguish discoloration originating from coating oxidation versus enzymatic browning occurring directly within the fruit tissue.

### 2.6 Statistical analysis

All experimental data were subjected to statistical analysis using SPSS software (version 25.0; SPSS Inc., Chicago, IL, USA). A two-way analysis of variance (ANOVA) was applied to evaluate the effects of treatments and storage time, as well as their interaction. Mean differences among treatments were assessed using Duncan’s multiple range test, with statistical significance established at *p* < 0.05. Principal component analysis (PCA) was carried out using SIMCA software (version 14.1; MKS Umetrics AB) to explore multivariate variations among FCA treatments (NT, CT, UC, OC, and UOC) over the storage period (0, 7, 14, 21, 28, 35, and 42 days). Furthermore, Pearson’s correlation analysis was performed to determine the relationships among the measured quality attributes during storage.

## 3 Results and discussions

### 3.1 Weight loss and firmness

It is common for fresh-cut produce to experience weight loss, wilting, and a reduction in firmness due to moisture evaporation, increased respiration rate, and cell wall degradation after cutting [5], [6], [61]. The weight loss and firmness values of FCA during storage are presented in Table 1. Weight loss gradually increased across all samples over time, with a significantly greater loss (17.41%) observed in the control (NT) by day 14 indicating rapid dehydration and tissue deterioration in the absence of protective barriers. In contrast, CT showed the lowest weight loss (4.58%) even after 42 days, followed by UOC (12.30%), demonstrating the strong moisture-retention ability. The coating effectively reduced transpiration and respiration-associated water loss by forming a protective barrier (semi-permeable) that limited oxygen transfer and moisture diffusion [9], [31]. The superior performance of CT further indicates that preservation of tissue integrity and maintenance of the natural cellular water-holding structure were more critical for long-term quality retention than only achieving initial microbial reduction. The incorporation of *S. platensis* and turmeric extracts likely enhanced this protective effect by providing antioxidant and antimicrobial functionality, thereby reducing oxidative membrane damage and delaying structural collapse during storage. Similar barrier-mediated reductions in weight loss have recently been reported in FCA and other fresh-cut produce treated with active edible coatings and coating-assisted hurdle preservation systems [9], [38], [58]. Although UOC also showed substantial protection against weight loss, its effectiveness remained slightly lower than CT. This difference may be associated with mild structural perturbation induced during the initial US and OZ treatments. US-induced cavitation and OZ-mediated oxidative reactions can temporarily increase membrane permeability and cellular stress, which may facilitate moisture migration during prolonged storage if not fully compensated by the coating matrix [55], [56]. Nevertheless, the lower weight loss observed in UOC compared with UC and OC suggests that combining mild non-thermal pretreatments with the active coating helped balance the beneficial microbial reduction effects while minimizing excessive tissue damage. The similar findings were documented by other researchers who showed that fresh-cut kiwifruit and cucumbers treated with a combination of coating and US experienced less reduction in weight [6], [39]. On the contrary, a study by Koushesh Saba et al. [31] reported no significant changes in weight for FCA treated with a combination of edible coatings containing ascorbic acid and CMC.

**Table 1:** Weight loss, firmness, decay incidence and ascorbic acid of fresh-cut apples (FCA) sample treatments (NT-UOC) from 0 to 42 days of storage.

**Storage time (Days)**	**Treatments**	**Weight Loss** **(%)**	**Firmness** **(N)**	**Decay Incidence (%)**	**Ascorbic acid** **(mg/g)**
**0**	**NT**	0	14.49 ± 0.25^a^	0	0.23 ± 0.02^f-i^
	**CT**	0	14.76 ± 0.14^a^	0	0.23 ± 0.03^f-i^
	**UC**	0	10.44 ± 0.42^i^	0	0.30 ± 0.04^a-d^
	**OC**	0	11.66 ± 0.50^g^	0	0.26 ± 0.03^b-f^
	**UOC**	0	12.55 ± 0.11^e^	0	0.32 ± 0.04^a^
**7**	**NT**	10.24 ± 0.14^e^	7.35 ± 0.16*^m^*	41.34^f^	0.12 ± 0.03^l^
	**CT**	1.45 ± 0.13*^n^*	14.12 ± 0.10^b^	0	0.24 ± 0.03^e-i^
	**UC**	3.31 ± 0.19^l^	8.41 ± 0.44^k^	0	0.31 ± 0.03^abc^
	**OC**	4.78 ± 0.13^j^	10.76 ± 0.11*^h^*	0	0.27 ± 0.02^a-f^
	**UOC**	1.11 ± 0.08^o^	12.15 ± 0.11^f^	0	0.30 ± 0.03^abc^
**14**	**NT**	17.41 ± 0.29^a^	1.35 ± 0.20^q^	100^a^	0.05 ± 0.03*^m^*
	**CT**	2.77 ± 0.15*^m^*	13.98 ± 0.11^b^	0	0.21 ± 0.03^g-k^
	**UC**	6.49 ± 0.16^h^	7.67 ± 0.15^l^	19.05^j^	0.31 ± 0.02^ab^
	**OC**	5.23 ± 0.14^i^	9.23 ± 0.14^j^	0	0.25 ± 0.04^d-h^
	**UOC**	3.31 ± 0.19^l^	11.75 ± 0.12^g^	0	0.28 ± 0.03^a-e^
**21**	**NT**	N/A	N/A	N/A	N/A
	**CT**	3.23 ± 0.17^l^	13.56 ± 0.15^c^	0	0.21 ± 0.03^g-k^
	**UC**	11.77 ± 0.14^d^	5.31 ± 0.12*^n^*	33.56^g^	0.21 ± 0.03^g-k^
	**OC**	9.27 ± 0.14^f^	7.26 ± 0.20*^m^*	26.59^h^	0.21 ± 0.04^g-k^
	**UOC**	6.28 ± 0.19^h^	10.79 ± 0.15^h^	0	0.26 ± 0.04^b-f^
**28**	**NT**	N/A	N/A	N/A	N/A
	**CT**	3.38 ± 0.18^l^	13.55 ± 0.08^c^	0	0.21 ± 0.03^g-k^
	**UC**	17.29 ± 0.17^a^	3.26 ± 0.17^p^	55.78^d^	0.11 ± 0.03^l^
	**OC**	14.31 ± 0.13^b^	4.82 ± 0.14^o^	46.23^e^	0.13 ± 0.02^l^
	**UOC**	7.25 ± 0.14^g^	10.29 ± 0.16^i^	4.76^l^	0.21 ± 0.02^g-k^
**35**	**NT**	N/A	N/A	N/A	N/A
	**CT**	3.68 ± 0.21^k^	13.18 ± 0.09^d^	0	0.20 ± 0.03^ijk^
	**UC**	N/A	N/A	78.21^b^	N/A
	**OC**	N/A	N/A	59.40^c^	N/A
	**UOC**	9.20 ± 0.19^f^	8.99 ± 0.15^j^	14.27^k^	0.18 ± 0.02^jk^
**42**	**NT**	N/A	N/A	N/A	N/A
	**CT**	4.58 ± 0.16^j^	12.75 ± 0.13^e^	0	0.17 ± 0.03^k^
	**UC**	N/A	N/A	100^a^	N/A
	**OC**	N/A	N/A	100^a^	N/A
	**UOC**	12.30 ± 0.19^c^	8.62 ± 0.22^k^	22.27^i^	0.19 ± 0.04^ijk^

Data are expressed as means ± SD and values with different superscript letters in a column differ significantly (*p* < 0.05).

Firmness followed a trend comparable to weight loss, decreasing progressively in all samples during storage because of moisture loss, enzymatic degradation of cell wall polysaccharides, and membrane disintegration. The most severe firmness reduction (90.68%) occurred in NT, where firmness declined from 14.49 N to 1.35 N by day 14 because of uncontrolled senescence and tissue breakdown. In contrast, CT maintained firmness remarkably well, showing only a 13.63% reduction over 42 days. This improved textural stability suggests that the coating effectively slowed degradation of pectic substances and reduced the activity of cell wall-degrading enzymes by limiting oxygen availability and oxidative stress. Recent studies have similarly reported that active coating systems preserve firmness by maintaining cell turgor pressure, reducing dehydration, and suppressing enzymatic softening reactions in fresh-cut fruits [9], [38]. UC and OC treatments initially reduced firmness immediately after treatment, with values of 10.44 N and 11.66 N, respectively, which suggests that US cavitation and OZ-induced oxidative reactions produced transient structural perturbation in the FCA tissues during the initial processing stage. US treatment can generate localized shear forces and microstreaming effects capable of partially disrupting cellular membranes and middle-lamella integrity, while OZ exposure may temporarily increase membrane permeability because of its highly reactive oxidative nature. However, despite this early softening effect, both treatments subsequently slowed further firmness deterioration during storage compared with NT, likely because microbial proliferation, oxidative degradation, and enzymatic tissue breakdown were effectively suppressed after treatment [60], [61]. UOC exhibited the best firmness retention among the non-thermal treatments (12.55 N at day 0), suggesting that combining shorter US and OZ exposure with the active coating minimized excessive tissue disruption while still providing microbial and oxidative protection [2], [40], [41]. Our findings align with the results of other studies on firmness of fresh-cut fruits during storage treated with edible coatings alone or in combination with non-thermal treatments [3], [6], [9], [31], [39], [42].

### 3.2 Decay incidence

Decay incidence in fresh-cut produce represents the progressive quality deterioration driven by moisture loss, enzymatic browning reactions, and microbial activity. The decay incidence of FCA treatments during storage, as shown in Table 1, indicates that the quickest spoilage occurred in NT samples, with a decay incidence of 41.34% at day 7 and complete spoilage (100%) by day 14. At this point, all other treated FCA remained decay-free except for UC, which showed a 19.05% decay incidence in samples. By day 21, decay in OC samples also increased, though slightly less than in UC, gradually reaching 78.21% in UC and 59.40% in OC by day 35. Both treatments resulted in complete spoilage (100%) by day 35. In contrast, UOC showed only 22.27% decay by day 42, while CT exhibited no signs of decay throughout the storage period. The rapid spoilage in NT was due to the absence of protective treatments, leading to uncontrolled microbial growth and enzymatic degradation. The improved preservation observed in CT can be attributed to the formation of a protective semi-permeable coating barrier that reduced oxygen diffusion, moisture loss, and external microbial contamination while simultaneously providing antioxidant and antimicrobial protection through the incorporated *S. platensis* and turmeric extracts [20]. UC and OC treatments delayed deterioration compared with the untreated control, however, their long-term preservation efficacy remained lower than CT and UOC. This reduced effectiveness may be associated with mild structural disruption and increased cellular stress induced during the initial treatment stages, which could have increased tissue susceptibility to subsequent oxidative deterioration during prolonged storage [60], [61]. Previous studies have reported that excessive or prolonged US exposure may partially disrupt cellular structures and membrane integrity, thereby accelerating quality degradation if processing conditions are not carefully optimized [55]. Similarly, OZ, despite its antimicrobial effectiveness, may induce oxidative stress responses and membrane perturbation in plant tissues because of its highly reactive oxidative nature, particularly when exposure conditions are not fully optimized [56]. In contrast, UOC showed significantly reduced decay, likely due to the combined action of US, OZ, and the active coating, which improved microbial control and maintained tissue integrity [59].

### 3.3 Ascorbic acid

Ascorbic acid slows oxidation, minimizes browning, and helps preserve freshness during storage. Ascorbic acid decreases during storage due to oxidation, enzymatic degradation, and light/temperature sensitivity [2], [43]. The greatest reduction (79%) was observed in NT, where levels decreased from 0.23 to 0.05 mg/g between day 0 and day 14. In contrast, the lowest reduction (26%) was recorded in actively coated CT samples, where ascorbic acid decreased from 0.23 to 0.17 mg/g over 42 days **(**Table 1**)**. This superior preservation suggests that the active edible coating effectively reduced oxygen diffusion and oxidative stress while limiting enzymatic oxidation reactions through its semi-permeable barrier properties. In addition, the antioxidant compounds present in *S. platensis* and turmeric extracts likely contributed to scavenging ROS and stabilizing endogenous vitamin C during storage. Tosif et al. [38] also demonstrated that a composite coating of aloe vera gel and CMC effectively slowed the reduction of ascorbic acid in FCA compared to the control during storage. UC and OC initially showed slightly increased or maintained ascorbic acid levels during early storage, likely because US cavitation and ozone exposure promoted temporary cellular permeability and release of intracellular compounds into the tissue matrix. However, these treatments subsequently exhibited faster degradation after day 21–28, coinciding with progressive spoilage and structural deterioration. This suggests that although non-thermal treatments initially reduced microbial load and delayed oxidative deterioration, excessive membrane perturbation and oxidative stress may have accelerated vitamin C degradation during prolonged storage when protective effects weakened. The UOC treatment exhibited a gradual 40% decrease from day 0 to day 42. The results indicate that active coatings, non-thermal treatments, and their combination effectively slowed the degradation of ascorbic acid. The active coating acted as a strong barrier, minimizing oxidation and enzymatic degradation, which helped preserve ascorbic acid in CT and non-thermally treated FCA. Meanwhile, non-thermal treatments induced an antioxidant defense mechanism, reducing oxidative stress, enzymatic activity, and microbial growth [60], [61]. However, as spoilage progressed, degradation accelerated. Likewise, Koushesh Saba et al. [31] reported comparable results related to FCA, showing that after 14 days of storage, ascorbic acid levels decreased from 13 to 1 mg/100 g in the control, from 13 to 4.20 mg/100 g in US treated samples, and from 13 to 10.58 mg/100 g in samples treated with a combined US and composite coating of CMC and ascorbic acid. Additionally, the study Zambrano-Zaragoza et al. [42] found that in fresh-cut cucumbers stored for 16 days, ascorbic acid decreased from 6 to 1.8 mg/100 g in the control, whereas the lowest reduction (from 6 to 5 mg/100 g) was observed in samples treated with UV-C and then coated with a hydroxypropyl methylcellulose-based active coating containing nano-encapsulated lemon peel essential oil.

### 3.4 Color parameters and browning index (BI)

Color parameters (L*, a*, b*) play a crucial role in evaluating the visual quality and consumer acceptance of fresh-cut produce, as they provide objective measurements of color changes, including browning [4], [11], [40]. Monitoring these values helps to evaluate the effectiveness of preservation treatments in maintaining fresh appearance and delaying browning. The color parameters (L*, a*, b*) and BI of FCA during storage are presented in Table 2. Color changes were observed across all treatments; however, compared to the NT, all other treatments effectively minimized the decrease in L* and b* values while controlling the increase in a* values. In the NT group, L* decreased significantly from 83.55 to 35.45, b* decreased from 28.32 to 14.67, a* increased from 1.65 to 18.44 and BI increased from 41.76 to 89.12% from day 0 and to day 14. The lowest color variations were observed in the CT treatment, where L* decreased from 82.34 to 54.69, b* from 27.35 to 19.71, a* increased from 1.84 to 4.39 and BI increased from 41.11 to 52.76% from day 0 to day 42 of storage. CT samples, treated only with the novel active coating, retained color better than other treatments because the coating formed a perfect semi-permeable barrier that reduced oxygen diffusion and moisture loss, thereby limiting enzymatic browning and microbial growth [31]. In line with the findings of Tosif et al. [38], where the aloe vera and CMC composite coating delayed the color changes in FCA during 10 days of storage. Similarly, UOC provided comparable results, while UC and OC also effectively delayed color changes, demonstrating their efficacy in preserving the visual quality of FCA. As both US and OZ helped in microbial inactivation and enzyme deactivation before coating application, which enhanced protection. Song et al. [11] also observed fewer changes in color of fresh-cut carrots treated with a combined US and citral nanoemulsion during 8 days of storage. In another study performed by Zambrano-Zaragoza et al. [42] fresh-cut cucumber demonstrated a similar trend of delay in color changes by the application of combined UV-C and edible nano-coating in 16 days of storage. Although the applied treatments reduced PPO activity and delayed overall browning development, gradual surface discoloration was still observed on coated FCA, particularly after day 21 of storage. Interestingly, when a thin superficial layer was removed, the underlying tissue appeared comparatively fresh, suggesting that the observed discoloration may have originated partially from oxidative or structural changes within the coating matrix rather than exclusively from enzymatic browning of the apple tissue itself. However, the present study did not include specific experiments designed to distinguish coating-associated discoloration from browning occurring directly in the fruit tissue, such as coating removal prior to color analysis or independent characterization of coating oxidation behavior. Therefore, the contribution of coating matrix oxidation versus tissue enzymatic browning could not be definitively separated and should be interpreted cautiously. Possible mechanisms contributing to the observed discoloration include oxidation of coating constituents, non-enzymatic browning reactions, moisture-loss-induced structural changes, and interactions between coating components and environmental oxygen during prolonged refrigerated storage. Nevertheless, the comparatively lower PPO activity, improved antioxidant retention, reduced oxidative stress markers, and better preservation of internal tissue quality collectively suggest that the treatments contributed substantially to delaying enzymatic deterioration of FCA.

**Table 2:** Browning index and color parameters of fresh-cut apples (FCA) sample treatments (NT-UOC) from 0 to 42 days of storage.

**Storage time (Days)**	**Treatments**	**Browning Index** **(%)**	**Color values**		
			L*	a*	b*
**0**	**NT**	41.76 ± 0.11^hi^	83.55 ± 0.23^d^	1.65 ± 0.14^kl^	28.32 ± 0.19^d^
	**CT**	41.11 ± 0.09^hi^	82.34 ± 0.21^e^	1.84 ± 0.06^k^	27.35 ± 0.17^f^
	**UC**	43.70 ± 0.11^fg^	86.71 ± 0.11^b^	0.38 ± 0.23^o^	31.51 ± 0.26^a^
	**OC**	40.89 ± 0.08^hi^	87.43 ± 0.14^a^	0.96 ± 0.10*^n^*	29.78 ± 0.14^b^
	**UOC**	42.31 ± 0.09^gh^	84.82 ± 0.15^c^	1.25 ± 0.18*^m^*	29.55 ± 0.12^b^
**7**	**NT**	52.33 ± 0.12^d^	61.42 ± 0.20^o^	7.54 ± 0.12^c^	21.42 ± 0.18*^n^*
	**CT**	42.59 ± 0.11^gh^	77.35 ± 0.20^i^	2.33 ± 0.13^j^	26.20 ± 0.18^h^
	**UC**	45.47 ± 0.09^f^	78.37 ± 0.13^h^	1.38 ± 0.19^lm^	28.58 ± 0.19^c^
	**OC**	44.78 ± 0.13^fg^	81.68 ± 0.15^f^	1.79 ± 0.11*^k^*	27.79 ± 0.21^e^
	**UOC**	43.94 ± 0.09^fg^	80.17 ± 0.20^g^	1.66 ± 0.21^kl^	28.30 ± 0.26^d^
**14**	**NT**	89.12 ± 0.13^a^	35.45 ± 0.21^v^	18.44 ± 0.32^a^	14.67 ± 0.22^r^
	**CT**	43.24 ± 0.11^fg^	68.37 ± 0.18^l^	2.40 ± 0.22^j^	23.31 ± 0.19^l^
	**UC**	54.18 ± 0.14^c^	65.31 ± 0.17*^m^*	5.62 ± 0.40^e^	25.27 ± 0.14^ij^
	**OC**	48.81 ± 0.12^e^	72.39 ± 0.17^k^	3.25 ± 0.18^i^	26.47 ± 0.13^g^
	**UOC**	47.88 ± 0.09^ef^	74.49 ± 0.18^j^	3.32 ± 0.19^i^	27.29 ± 0.17^f^
**21**	**NT**	N/A	N/A	N/A	N/A
	**CT**	45.00 ± 0.12^f^	63.42 ± 0.18*^n^*	2.46 ± 0.15^j^	22.22 ± 0.20*^m^*
	**UC**	58.00 ± 0.11^b^	63.52 ± 0.09*^n^*	7.66 ± 0.23^c^	25.04 ± 0.14^j^
	**OC**	54.65 ± 0.09^c^	65.41 ± 0.16*^m^*	4.03 ± 0.14^h^	26.26 ± 0.07^gh^
	**UOC**	49.24 ± 0.16^e^	68.32 ± 0.19^l^	3.41 ± 0.20^i^	25.47 ± 0.15^i^
**28**	**NT**	N/A	N/A	N/A	N/A
	**CT**	47.76 ± 0.13^ef^	58.87 ± 0.13^q^	3.23 ± 0.14^i^	21.22 ± 0.18*^n^*
	**UC**	59.82 ± 0.15^b^	51.30 ± 0.17^u^	9.36 ± 0.13^b^	19.29 ± 0.16^q^
	**OC**	59.41 ± 0.12^b^	53.40 ± 0.25^t^	6.73 ± 0.26^d^	21.39 ± 0.17*^n^*
	**UOC**	54.12 ± 0.16^c^	59.35 ± 0.21^p^	4.31 ± 0.14^gh^	23.67 ± 0.15^k^
**35**	**NT**	N/A	N/A	N/A	N/A
	**CT**	49.71 ± 0.16^e^	55.32 ± 0.18^r^	4.23 ± 0.19^gh^	21.16 ± 0.13*^n^*
	**UC**	N/A	N/A	N/A	N/A
	**OC**	N/A	N/A	N/A	N/A
	**UOC**	54.12 ± 0.11^c^	55.38 ± 0.16^r^	4.28 ± 0.19^gh^	23.24 ± 0.11*^l^*
**42**	**NT**	N/A	N/A	N/A	N/A
	**CT**	52.76 ± 0.15^d^	54.69 ± 0.13^s^	4.39 ± 0.20^fg^	19.71 ± 0.17^p^
	**UC**	N/A	N/A	N/A	N/A
	**OC**	N/A	N/A	N/A	N/A
	**UOC**	58.88 ± 0.15^b^	51.47 ± 0.18^u^	4.61 ± 0.14^f^	20.41 ± 0.16^o^

Data are expressed as means ± SD and values with different superscript letters in a column differ significantly (*p* < 0.05).

### 3.5 pH, total soluble solids (TSS), and titratable acidity (TA)

**Fig. 2:**
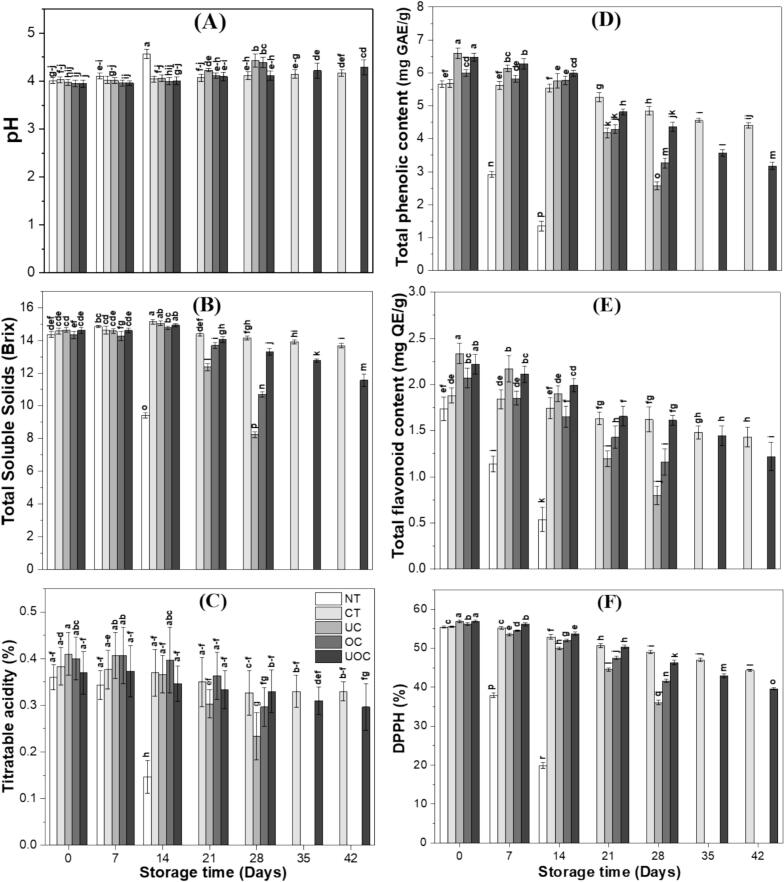
(A) pH, (B) TSS, (C) TA, (D) TPC, (E) TFC, and (F) Antioxidant activity by DPPH of fresh-cut apples (FCA) sample treatments (NT-UOC) from 0 to 42 days of storage. Treatments with different letters above bars show significant differences (*p* < 0.05).

pH, TSS, and TA are key indicators of freshness, flavor, and quality in FCA, influencing taste, texture, and microbial stability during storage. The changes in pH, TSS, and TA of apple samples during storage are shown in Fig. 2. The active coating, both alone and in combination with US, OZ, and their combination, effectively stabilized the pH compared to the control (NT) during storage. In NT, the pH increased significantly (*p* < 0.05) from 4.01 to 4.57 within 14 days, indicating rapid spoilage. This increase may be associated with degradation of organic acids, microbial metabolism, and accumulation of alkaline by-products during advanced spoilage stages. In contrast, the lowest pH increase was observed in CT, where it rose gradually from 4.03 to 4.17 within 42 days (Fig. 2**(A)**). The improved pH stability observed in CT may be attributed to the active coating reducing oxygen exposure, respiration rate, oxidative deterioration, and microbial proliferation through its semi-permeable barrier properties [2]. TSS initially increased in all samples before declining as spoilage set in. The initial increase in TSS can be attributed to the enzymatic breakdown of polysaccharides into simpler sugars, while the subsequent decline is likely due to microbial consumption and fermentation [13]. In NT, TSS rose from 14.35°Brix at day 0 to 14.85°Brix at day 7 before significantly (*p* < 0.05) dropping to 9.42°Brix by day 14. However, in CT, TSS increased from 14.58°Brix at day 0 to 15.14°Brix at day 14 and then gradually declined to 13.69°Brix only by day 42, demonstrating the protective effect of the active coating. These findings suggest that the active coating effectively delayed metabolic degradation and respiration-associated sugar consumption by limiting oxygen availability and microbial growth. A similar pattern was observed in UC and OC until day 21, followed by a significant decrease at day 28. In UOC, TSS declined from 14.62°Brix at day 0 to 11.57°Brix by day 42 (Fig. 2**(B)**). In UOC, the combined effects of mild US and OZ pretreatments together with coating protection likely slowed microbial spoilage and oxidative deterioration while minimizing excessive tissue stress, thereby improving long-term biochemical stability compared with UC and OC alone.

TA exhibited an opposite trend to pH, decreasing progressively during storage because organic acids are continuously utilized as respiratory substrates and metabolized during enzymatic and microbial activities [20]. In NT, TA increased from 0.36% to 0.15% between day 0 and day 14. In comparison, TA in CT decreased from 0.38% to 0.33%, while in UOC, it declined from 0.37% to 0.30% over 42 days, indicating significant prevention of acidity loss (Fig. 2**(C)**). The active coating, alone or in combination with non-thermal treatments (US, OZ, US + OZ), slowed these changes by reducing microbial load, minimizing enzymatic activity, and forming a protective barrier that delayed oxidation and moisture loss, thereby maintaining overall quality. Similar findings related to changes in pH, TSS and TA have been obtained by studies of Tosif et al. [38], Nicolau-Lapeña et al. [9], Koushesh Saba et al. [31], and Pandey et al. [44] performed to evaluate the effects of active coatings and their combination with US on extending the shelf life of apples.

### 3.6 Total phenolic content (TPC), total flavonoid content (TFC), and antioxidant activity

TPC and TFC are key indicators of antioxidant capacity, but during storage they decreased due to oxygen exposure, enzymatic oxidation, and microbial activity as shown in Fig. 2 **(D)** and **(E)** [42]. This reduction in TPC and TFC diminishes the antioxidant activity (Fig. 2 **(F)**), leading to increased oxidative damage and accelerated spoilage. As expected, the highest decrease in these parameters was observed in NT, where TPC decreased by 76.1% (from 5.65 to 1.35 mg GAE/g), TFC decreased by 69.0% (from 1.74 to 0.54 mg QE/g), and DPPH % inhibition declined from 55.40% to 19.90% (a 64.1% reduction) from day 0 to 14. All other treatments significantly reduced the loss of bioactive compounds, with the best results observed in CT, where TPC decreased by 22.2% (from 5.67 to 4.41 mg GAE/g), TFC decreased by 23.9% (from 1.88 to 1.43 mg QE/g), and DPPH % inhibition declined from 55.52% to 44.37% (20.1% reduction) from day 0 to 42. The active coating acted as physical barrier reducing the permeability of oxygen and oxidative agents to the apple tissue which slowed down the enzymatic browning and it also provided antimicrobial action by releasing bioactive compounds that helped to minimze the loss of TPC and TFC. In contrast, UC, OC, and UOC, despite their initial benefits in enhancing bioactive extractability and microbial reduction [60], [61], may have caused microstructural damage to the apple tissue [55], [56]. This damage increased the exposure of internal tissues to oxygen and oxidative enzymes, accelerating degradation over time that is why UC and OC better retained these bioactives only until day 14. The effectiveness of UOC was similar to CT due to the synergistic combination of US and OZ enhancing antioxidative and antimicrobial effects, while the shorter treatment times minimized structural damage, allowing the coating to provide continuous protection and slow bioactive compound loss effectively. Similar synergistic preservation effects of coating-assisted non-thermal hurdle systems have recently been reported in FCA, cucumbers, and cantaloupe, where combined treatments improved antioxidant retention and delayed oxidative spoilage more effectively than individual preservation approaches [7], [31], [42], [45], [46].

### 3.7 Hydrogen peroxide (H_2_O_2_) content, superoxide anions (O_2_^−^) production rate and malondialdehyde (MDA) content

**Fig. 3:**
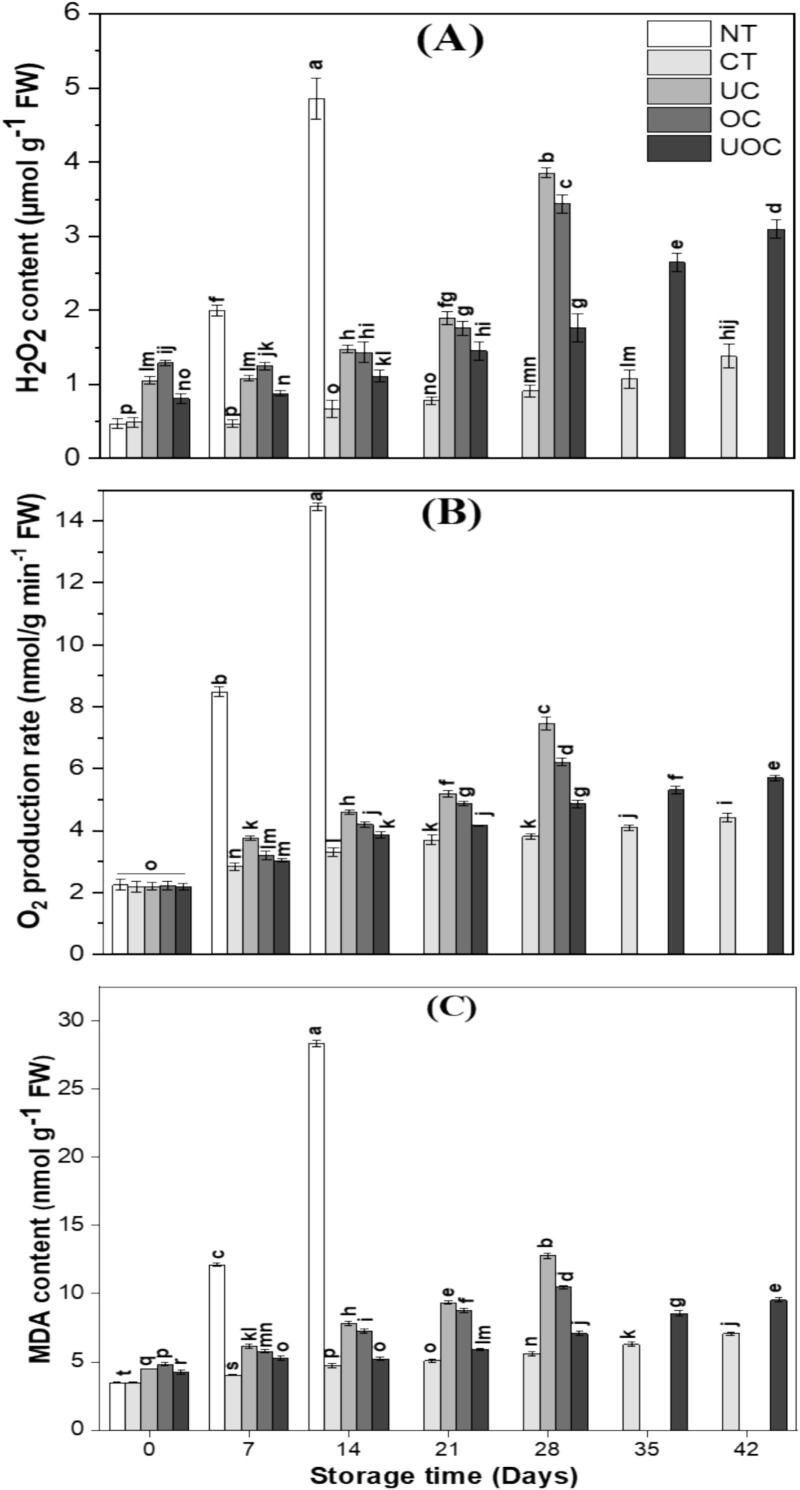
(A) Hydrogen peroxide (H_2_O_2_) content, (B) Superoxide anions (O_2_^−^) production rate, and (C) Malondialdehyde (MDA) content of fresh-cut apples (FCA) sample treatments (NT-UOC) from 0 to 42 days of storage. Treatments with different letters above bars show significant differences (*p* < 0.05).

Free radicals are produced and removed in a stable manner in fresh produce, playing a crucial role in cell signaling and homeostasis. However, this balance is disrupted when damage occurs, such as during fresh-cut processing, leading to the overproduction of free radicals like H_2_O_2_ and O_2_^−^. Additionally, lipid peroxidation catalyzed by LOX enzymes produces MDA, a marker of oxidative stress [20]. The oxidative damage caused by these species accelerate cell membrane damage, increase permeability and senescence, resulting in the rapid spoilage. As shown in Fig. 3 **(A)** and **(B)**, OC exhibited the highest H_2_O_2_ and MDA levels immediately after treatment at day 0, followed by UC and UOC, indicating that OZ and US treatments initially induced oxidative and structural stress in the apple tissues. OZ exposure may temporarily increase ROS accumulation because of its highly reactive oxidative nature, while US cavitation can induce transient membrane perturbation and localized oxidative stress through microstreaming and shear effects [55], [56]. However, despite this initial increase, oxidative stress markers remained relatively controlled in UC and OC until day 21 and in UOC until day 35, suggesting that the treatments still delayed subsequent deterioration compared with NT. In NT, the initial levels of H_2_O_2_ content (0.47 µmol g^−1^) and MDA content (3.48nmol g^−1^) at day 0 were lower than those in UC, OC, and UOC, and were similar to CT. However, these levels, along with the O_2_^−^ production rate (2.25 nmol/g min^−1^) (Fig. 3 **(C)**), increased sharply to H_2_O_2_ content (4.86 µmol g^−1^), O_2_^−^ production rate (14.46 nmol/g min^−1^), and MDA content (28.34nmol g^−1^) by day 14, when NT samples spoiled completely, due to the absence of protective treatments. This rapid accumulation indicates severe oxidative deterioration resulting from uncontrolled respiration, microbial proliferation, membrane degradation, and enzymatic oxidation in untreated tissues. In contrast, CT showed the lowest increase, with H_2_O_2_ content rising from 0.49 µmol g^−1^ to 1.38 µmol g^−1^, O_2_^−^ production rate from 2.19 nmol/g min^−1^ to 4.42 nmol/g min^−1^, and MDA content from 3.47nmol g^−1^ to 7.08nmol g^−1^ from day 0 to day 42, as the active coating effectively reduced oxidative stress and enzymatic activity. In UOC, the values were slightly higher than CT at day 42 but showed only minor signs of spoilage, demonstrating the synergistic effects of combining US, OZ, and the coating in prolonging shelf life. These results are consistent with the findings of Xin et al. [1], who demonstrated that a whey protein-based coating effectively controlled H_2_O_2_ and MDA content in FCA during 8 days of storage compared to non-coated samples, where a sharp increase was observed. Similarly, Yu et al. [47] reported the lowest increase in H_2_O_2_ content and O_2_^−^ production rate in fresh-cut asparagus lettuce coated with CMC containing ascorbic acid and L-cysteine compared to non-coated samples during 16 days of storage. In another study, Ali et al. [48] also found a significantly lower increase in H_2_O_2_ content, O_2_^−^ production rate, and MDA content in aloe vera gel-coated lotus root slices compared to non-coated samples over 8 days of storage.

### 3.8 Enzymatic activities

**Fig. 4:**
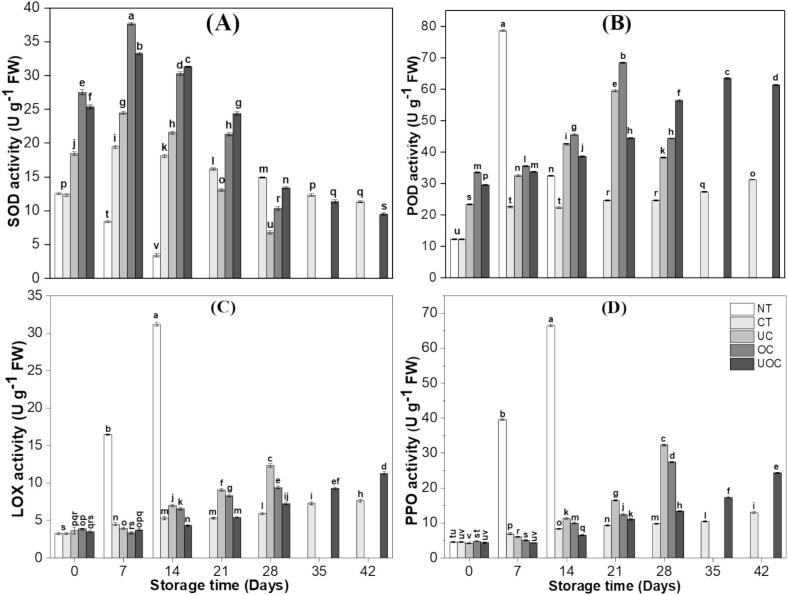
(A) Superoxide dismutase (SOD), (B) Peroxidase (POD), (C) Lipoxygenase (LOX) and (D) Polyphenol oxidase (PPO) of fresh-cut apples (FCA) sample treatments (NT-UOC) from 0 to 42 days of storage. Treatments with different letters above bars show significant differences (*p* < 0.05).

Enzymes play critical roles in determining the postharvest quality of FCA. As shown in Fig. 4 **(A)**, SOD activity exhibited distinct patterns across treatments. Initial activity was highest in OC (27.54 U g^−1^) at day 0, followed by UOC (25.38 U g^−1^), UC (18.49 U g^−1^), NT (12.52 U g^−1^) and CT (12.37 U g^−1^), reflecting the immediate oxidative stress response to US, OZ and US + OZ treatment [55], [56]. The activity peaked at 37.68 U g^−^1 in OC by day 7, with similar increases in other treatments except NT where it declined to 8.43 U g^−^1, indicating the system's collapse in untreated samples because of high spoilage. From day 7 onward, SOD activity gradually declined in all treatments, reaching lowest values at spoilage points (NT at day 14; UC/OC at day 21). Notably, CT showed initial activation at day 7 before slowly declining to baseline (Day 0) by day 42, suggesting sustained but moderate oxidative stress. UOC's SOD activity stabilized to CT levels by day 35, demonstrating that the combined treatment's protective effects eventually matched the coating alone. These patterns confirm that non-thermal treatments initially induced greater oxidative stress (higher SOD), while the coating provided more stable, long-term protection against free radicals. POD contributes to both antioxidant defense and undesirable browning reactions through its dual role in H_2_O_2_ metabolism [60]. Similar to SOD, OC showed the highest initial POD activity (33.55 U g^−^1 at day 0) followed by UOC, and UC, as non-thermal treatments generated H_2_O_2_ that required immediate enzymatic neutralization Fig. 4 **(B)**. NT exhibited a rapid increase in POD activity, reaching 78.73 U g^−1^ at day 7, followed by a sharp decline to 32.51 U g^−1^ at day 14. This pattern suggests severe oxidative stress and accelerated tissue deterioration, where initial enzyme induction was followed by progressive loss of enzymatic functionality because of advanced spoilage and membrane degradation. UC and OC maintained elevated POD activity until later storage stages, indicating continued oxidative stress pressure throughout storage. The combined treatment UOC displayed unique behavior with POD activity gradually increasing until day 35 and showing only minor spoilage by day 42, suggesting synergistic stress mitigation. Most significantly, CT maintained remarkably stable POD levels after an initial adaptive doubling by day 7, demonstrating the coating's dual protective mechanism, immediate enzymatic support followed by sustained barrier protection. Overall, the enzymatic activity patterns observed in CT and UOC demonstrate that active coating-assisted preservation effectively regulated oxidative stress responses and delayed enzymatic deterioration during refrigerated storage. Similar stabilization of antioxidant enzyme systems has been reported in fresh-cut fruits treated with edible coatings and coating-assisted non-thermal hurdle preservation technologies [31], [47], [48].

LOX drives lipid oxidation, leading to the development of rancid flavors and the accumulation of malondialdehyde, a key indicator of oxidative damage. While all treatments showed comparable initial LOX activity (OC highest at 3.88 U g^−1^), their trajectories diverged markedly during storage (Fig. 4 **(C)**). NT exhibited the most dramatic increase, peaking at 31.21 U g^−^1 by day 14 at complete spoilage, demonstrating uncontrolled lipid peroxidation. UC and OC samples showed minor LOX activation until day 21 but at day 28 highest activity was observed (12.35 and 9.42 U g^−^1 respectively), indicating gradual membrane degradation. Notably, the combined treatment (UOC) reached its highest LOX activity (11.32 U g^−^1) only at day 42 with minimal visible spoilage, suggesting an effective delay of lipid oxidation. Most impressively, the CT maintained consistently low LOX levels throughout storage with the highest value of 7.67 U g^−^1 only at day 42, demonstrating the coating's superior protection against membrane damage. The most notorious of these enzymes, PPO, directly catalyzes the browning reaction by oxidizing phenolic compounds, resulting in the unappealing discoloration in fres-cut produce that significantly reduces product marketability [1], [2], [60]. The PPO activity patterns reveal critical differences in browning control between treatments as shown in Fig. 4 **(D)**. While all samples started with similar baseline PPO levels, NT samples showed rapid enzymatic browning due to unimpeded phenolic oxidation, reaching 66.49 U g^−1^ PPO activity by day 14. UC and OC treatments demonstrated better PPO activity control, peaking at 32.34 and 27–32 U g^−1^ by day 28, as the non-thermal treatments partially inactivated PPO [31]. Notably, the UOC maintained good stability against PPO activity with the highest value (24.37 U g^−1^ PPO activity) obtained at day 42. The superior performance of CT (only 13.06 U g^−1^ PPO activity at day 42) highlights that coating provided superior oxygen barrier properties, direct inhibition of PPO activity and avoiding pretreatment cellular damage preserved natural compartmentalization of phenolic compounds and enzymes [9], [39]. Browning caused by PPO is a critical issue. While CT samples remained unspoiled at day 42 and UOC samples had minimal spoilage, significant color degradation was observed. Although active coating alone and in combination with non-thermal treatments significantly delayed browning, color degradation became a major concern, particularly after day 21. Overall, CT alone performed better because non-thermal pretreatments (UC/OC) caused cellular damage, increasing oxidative stress that overwhelmed the coating's protection, while CT's provided immediate, stable defense without prior harm. UOC mitigated this slightly by combining treatments synergistically but still couldn't match CT's long-term consistency. Our findings align with previous studies of Xin et al. [1] who reported that whey protein-based emulsion coatings controlled PPO activity in FCA during eight days of storage. Koushesh Saba et al. [31] found that SOD activity was highest and PPO activity was lowest in FCA treated with US and ascorbic acid + CMC coating, whereas the control showed the opposite trend by the eighth day of storage. Similarly, Fan et al. [39] observed a similar pattern of increased POD and PPO activity, with better enzymatic control achieved through carbon dot coatings alone and in combination with US in fresh-cut cucumber during 15 days of storage. The other studies have also provided similar results related to enzymatic activities [42], [47], [48].

### 3.9 Microbial analysis

**Fig. 5:**
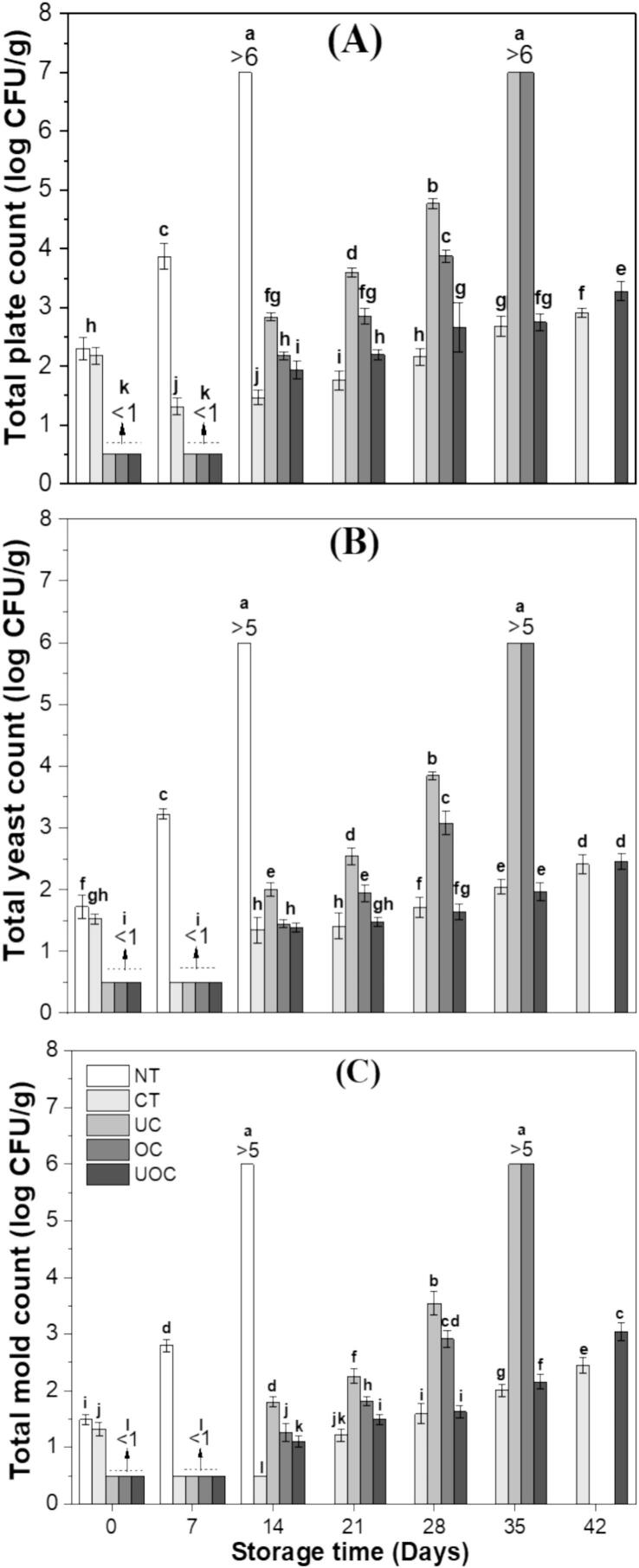
(A) Total plate count, (B) Total yeast count, and (C) Total mold count of fresh-cut apples (FCA) sample treatments (NT-UOC) from 0 to 42 days of storage. Treatments with different letters above bars show significant differences (*p* < 0.05).

The shelf life of fresh-cut produce is largely determined by microbial growth (Bacteria and fungi), which drives spoilage through biochemical degradation causing off-flavor development and texture degradation [60], [61]. All samples showed increasing microbial counts during storage, but at treatment-dependent rates (Fig. 5). Although NT and CT exhibited relatively similar initial microbial counts, their microbial growth patterns diverged markedly during storage. NT rapidly exceeded spoilage thresholds (>6 log CFU g^−^1 for total plate count and > 5 log CFU g^−^1 for yeast and mold) by day 14, indicating severe microbial deterioration in untreated tissues. This rapid increase likely resulted from unrestricted microbial proliferation combined with accelerated tissue degradation, moisture loss, and oxidative deterioration following fresh-cut processing. CT showed an initial microbial reduction (day 0–7: bacteria 2.18 → 1.31; yeast 1.52→<1; mold 1.33→<1 log CFU/g) because of the active coating's antimicrobial compounds. CT demonstrated superior microbial control throughout storage, with maximum counts at day 42 remaining well below spoilage thresholds (total plate count: 2.91 log CFU/g; yeast: 2.41 log CFU/g; mold: 2.45 log CFU/g). These low microbial levels explain the absence of spoilage in CT samples at day 42, which can be attributed to the physical barrier created by the active aloe vera coating matrix significantly reduced microbial attachment and penetration, gradual release of antimicrobial compounds from *S. platensis* and turmeric extracts provided continuous protection against bacterial and fungal growth, and coating's ability to maintain a modified atmosphere around the apple slices further inhibited microbial proliferation [9]. UC and OC samples maintained initial sterility (<1 log CFU/g) until day 7 due to the immediate antimicrobial effects of cavitation (physical disruption of microbial cells) and ozone (oxidative damage to cell membranes) [2], [60], [61]. While the applied active coating provided additional antimicrobial support, extending protection until day 28 but not upto 42 days. Its because the treatment-induced cellular damage at start created favorable conditions for subsequent microbial proliferation and this explains the complete spoilage observed by day 35, when counts exceeded thresholds (>6 log CFU/g bacteria; >5 log CFU/g yeast/mold) [2]. The results demonstrate that while non-thermal treatments achieve effective initial microbial reduction, their cellular damage and lack of residual activity limit long-term efficacy compared to the intact coating approach. UOC maintained initial microbial levels (<1 log CFU/g) through day 7, demonstrating effective early-stage microbial control from the US + OZ + active coating synergy. Microbial counts began appearing at day 14 but remained comparable to CT samples through day 35, because of less cellular damage due to mild parameters. However, by day 42, UOC showed slightly elevated counts (total plate: 3.28; yeast: 2.46; mold: 3.04 log CFU/g) versus CT, explaining its minor spoilage signs. This suggests that while the combined treatment delayed microbial recovery better than individual non-thermal methods resulting in enhanced shelf life, some damage to apple cells allowed limited microbial proliferation at later stages, though still far below the > 5–6 log CFU/g spoilage thresholds seen in UC and OC. The antimicrobial mechanism of the active coating and non-thermal treatments primarily involved disrupting microbial cell integrity by altering cell permeability, leading to leakage of intracellular contents. Additionally, diffusion of active compounds interacted with microbial DNA, inhibiting mRNA transcription and protein synthesis, ultimately preventing microbial growth [2]. The analogous findings have been documented by research conducted on utilization of active edible coatings alone and in combination with non-thermal treatments on FCA [5], [9], [31], green peppers [43], cucumber [10], [39], [42], carrots [11], and cantaloupes [2].

### 3.10 Overall visual acceptability

**Fig. 6:**
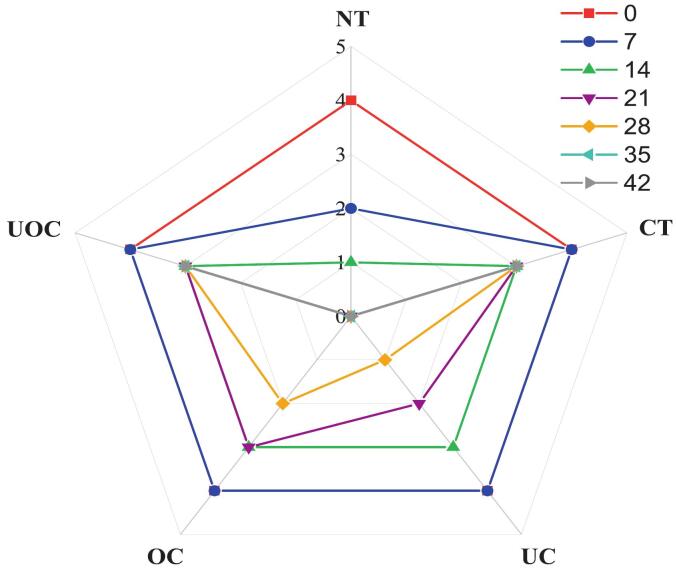
Changes in overall visual acceptability scores of fresh-cut apples (FCA) samples during storage. Visual acceptability was evaluated at 0, 7, 14, 21, 28, 35, and 42 days using a 4-point quality scoring system, where 4 = excellent quality, 3 = good quality, 2 = not saleable but still edible, and 1 = poor quality/spoiled. Treatments included NT (untreated control), CT (active edible coating only), UC (ultrasonication followed by active edible coating), OC (ozonation followed by active edible coating), and UOC (combined ultrasonication and ozonation followed by active edible coating). Values represent the average of three independent evaluations performed under standardized laboratory conditions.

**Fig. 7:**
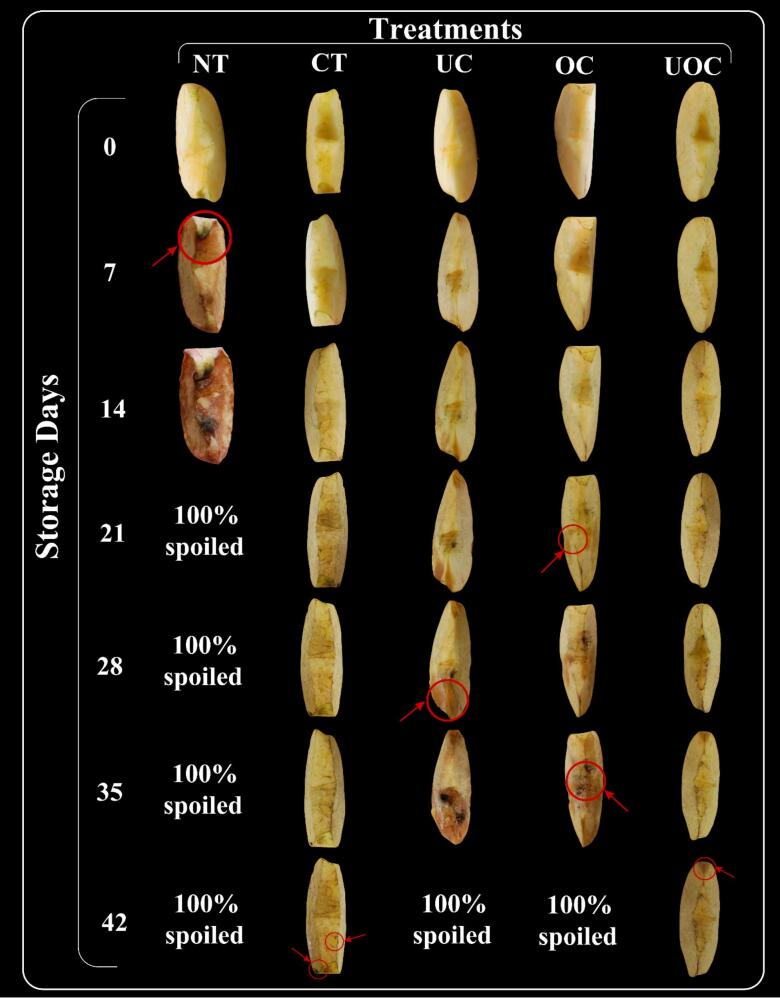
Visual appearance and surface discoloration changes of fresh-cut apples (FCA) samples during storage. Storage intervals included 0, 7, 14, 21, 28, 35, and 42 days. Treatments consisted of NT (untreated control), CT (active edible coating only), UC (ultrasonication followed by active edible coating), OC (ozonation followed by active edible coating), and UOC (combined ultrasonication and ozonation followed by active edible coating). Red indicators highlight representative regions showing browning, discoloration, and visible deterioration during storage.

Overall visual acceptability refers to the combined assessment of color, appearance, and freedom from any defect that determines whether FCA remain appealing to consumers throughout storage. The overall visual acceptability scores and visual appearance of the FCA varied significantly depending on the applied treatment, as clearly demonstrated in Fig. 6. NT showed the most rapid decline, becoming completely unacceptable by day 14 due to severe color changes, enzymatic browning and microbial spoilage. Among the treated samples, those subjected to UC became unacceptable by day 28, while OC maintained marginally better quality until day 35, reflecting the differential effectiveness of these non-thermal treatments. The highest and most stable acceptability was observed in both the CT and UOC, which maintained excellent scores of 4 points at day 14 and retained good scores of 3 points even at day 42. Although photographs shown in Fig. 7 shows gradual surface discoloration after day 21, the underlying apple tissue frequently appeared less deteriorated beneath the surface coating layer. These observations suggested that part of the discoloration may have been associated with oxidative or structural changes occurring within the coating matrix in addition to tissue enzymatic browning; however, the present study did not include specific experiments capable of definitively distinguishing between these phenomena. Therefore, interpretation of the visual appearance results should be considered with caution. Nevertheless, the comparatively lower PPO activity, reduced oxidative stress markers, improved firmness retention, and absence of severe microbial spoilage collectively support the protective effects of CT and UOC treatments during storage. The improved preservation performance of these treatments may be attributed to the combined barrier properties of the coating system and the antioxidant potential of the incorporated extracts, which likely contributed to reducing oxidative and enzymatic deterioration pathways. Overall, the results suggest that the active coating, particularly CT and UOC, substantially improved the storage stability and visual quality retention of FCA compared with untreated samples.

### 3.11 Multivariate statistical analysis of fresh-cut apples (FCA) postharvest quality during storage

**Fig. 8:**
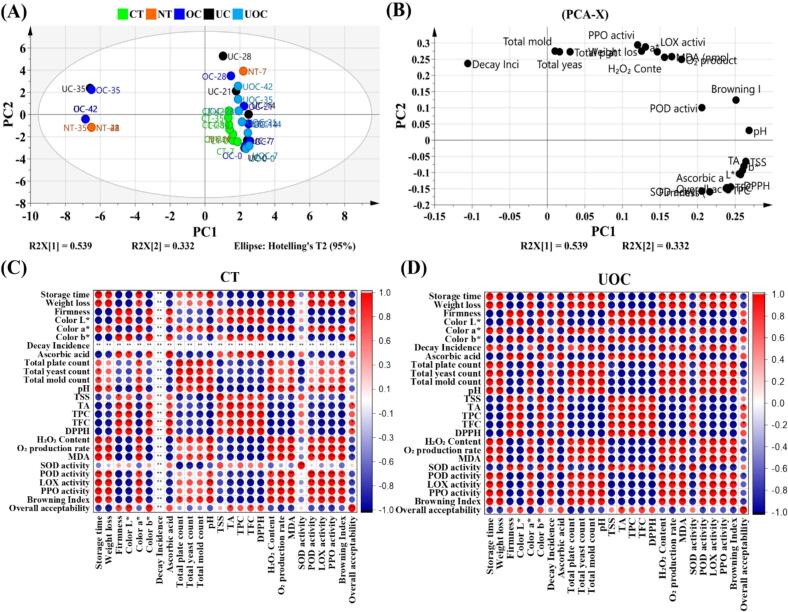
Multivariate statistical analysis of fresh-cut apples (FCA) qualities during storage. (A) and (B) principal component analysis (PCA) score plots. (C) and (D) Pearson’s correlation analysis matrix for the analyzed quality parameters of fresh-cut apple treatments CT and UOC, respectively. The correlation coefficient value is proportional to the color intensity and size of the circle. Negative and positive correlations are represented in blue and red colors, respectively. * Denotes significant differences among postharvest quality parameters (*p* < 0.05 (*), *p* < 0.01 (**)).

Fig. 8**(A)** displays the PCA score plot of FCA treatment samples (NT, CT, UC, OC and UOC) stored for 0, 7, 14, 21, 28, 35 and 42 days, while Fig. 8**(B)** shows the distribution of overall quality parameters in space defined by first and second PCA dimensions. Results showed that the first (PC1) and the second component (PC2) account for 87.1% of the total variation among FCA treatment samples. Of the total variation, 53.9% was attributed to the first PC1 and 33.2% to PC2 (Fig. 8**(A)**). As a result, samples separated by PC1 are more distinct from those separated by PC2. Furthermore, UOC and CT samples demonstrated a similar overall profile that differed significantly from the profiles of NT, UC, and OC samples, particularly on days 35 and 42. PC1 correlated positively with almost all quality parameters, including weight loss, firmness, pH, TA, color L*, color a*, color b*, MDA, PPO, POD, LOX activity, etc., except for the decay index. In contrast, PC2 was positively correlated with all examined quality parameters except for DPPH, SOD activity, TA, TSS, overall acceptability, b* values, TFC, TPC, ascorbic acid, and firmness (Fig. 8**(B)**).

On the other hand, the Pearson correlation coefficient shows the positive (in red) and negative (in blue) correlation between all the examined quality attributes of FCA during storage, as illustrated in Fig. 8**(C) and (D)**. Both CT and UOC treatments showed strong significant positive correlations between decay incidence and storage time, weight loss, pH, MDA, PPO, POD, total plate count, total yeast count, LOX activity, O_2_ production rate, a* values, and H_2_O_2_ content and browning index, implying that an increase in these quality parameters plays an important role in FCA decay. Furthermore, the decay incidence of CT and UOC treatments was significantly negatively correlated with firmness, L*, b*, ascorbic acid, TA, TSS, TPC, TFC, and DPPH, indicating that the decay incidence of FCA gradually increased as these parameters decreased. Conversely, CT revealed no relationship between the decay incidence and the other quality parameters of FCA. This is attributed to FCA treated with CT exhibiting no signs of decay throughout the storage period.

## 4 Future directions

One limitation of the present study was the inability to experimentally distinguish between discoloration originating from the coating matrix and enzymatic browning occurring directly within the apple tissue during prolonged refrigerated storage. Although the treatments preserved microbial, textural, nutritional, antioxidant, and overall physicochemical quality attributes, gradual surface discoloration remained evident after extended storage. Since visual appearance is a major determinant of consumer acceptance, future studies should focus on improving surface color retention and clarifying the mechanisms responsible for coating-associated discoloration. This may involve incorporating coating-only controls, evaluating coating oxidation behavior separately from fruit tissue responses, optimizing the formulation of the aloe vera–based coating, modifying the ratios of *S. platensis* and turmeric extracts, or incorporating additional natural anti-browning agents. Future investigations should also evaluate alternative or complementary non-thermal technologies capable of minimizing cellular stress while maintaining antimicrobial efficacy. Moreover, integration with modified atmosphere packaging, humidity regulation systems, or secondary protective coating layers may further reduce oxidative surface deterioration under commercial storage conditions. Finally, large-scale validation studies focusing on process scalability, economic feasibility, and industrial implementation are required to support the practical application of these sustainable preservation strategies in the fresh-cut produce industry.

## 5 Conclusion

An aloe-vera–based active edible coating enriched with *S. platensis* and turmeric extracts effectively maintained the physicochemical, microbiological, and functional quality of FCA during refrigerated storage. The coating applied alone (CT) consistently provided the most stable long-term preservation, limiting moisture loss, maintaining firmness, reducing oxidative stress and enzymatic browning, and preserving antioxidant compounds and microbial safety throughout storage. The combined treatment involving US and OZ followed by coating (UOC) showed similar preservation trends, suggesting that these non-thermal technologies may contribute to early-stage microbial reduction and improved coating dispersion; however, under the conditions applied in this study, they did not provide superior end-of-storage performance compared with the coating alone. The most important parameter color was better retained in CT and UOC, although surface browning was observed after day 21, with CT showing L* (54.69), a* (4.39), b* (19.71), and BI (52.76%) at day 42. Although some degree of surface discoloration developed after day 21, particularly under prolonged storage without modified atmosphere or additional anti-browning agents, this change did not reach the rejection threshold because texture, microbial quality, aroma, and overall eating quality remained acceptable. Multivariate analysis confirmed that CT and UOC preserved overall quality, while NT, UC, and OC deteriorated rapidly. In conclusion, the active edible coating alone provided superior long-term protection against weight loss, firmness reduction, microbial spoilage, oxidative damage, enzymatic browning, and antioxidant loss, while its combination with mild non-thermal treatments (UOC) also offered better preservation with minimal cellular damage, significantly extending the shelf-life of FCA up to 42 days compared to 14 days in the control. While UC and OC treatments caused initial cellular damage that limited their effectiveness to 28 days of preservation, this still represented a substantial improvement over existing methods documented in literature, which typically achieve only upto 23 days of shelf-life of FCA. However even CT and UOC preserved composite quality far better than the untreated control (which spoiled by day 14), the visual changes remain an area for improvement. These findings carry important practical implications for the fresh-cut produce industry, offering viable solutions to significantly extend the marketable life of apples and reduce postharvest losses that currently pose substantial economic and food security challenges.

## Declaration of generative AI in scientific writing

During the preparation of this work the authors used ChatGPT in order to improve readability and language. After using this tool, the authors reviewed and edited the content as needed and take full responsibility for the content of the publication.

## CRediT authorship contribution statement

**Samran Khalid:** Writing – review & editing, Writing – original draft, Methodology, Formal analysis, Data curation, Conceptualization. **Kashmala Chaudhary:** Writing – original draft, Visualization, Methodology, Formal analysis, Data curation, Conceptualization. **Ahmed Fathy Ghazal:** Writing – review & editing, Visualization, Formal analysis. **Abderrahmane Aït-Kaddour:** Writing – review & editing, Formal analysis. **Marwa Ezz El-Din Ibrahim:** Writing – review & editing, Funding acquisition, Formal analysis. **Rana Muhammad Aadil:** Writing – review & editing, Supervision, Methodology.

## Funding

This work was supported by the Deanship of Scientific Research, Vice Presidency for Graduate Studies and Scientific Research, King Faisal University, Saudi Arabia [GRANT: KFU261320].

## Declaration of competing interest

The authors declare that they have no known competing financial interests or personal relationships that could have appeared to influence the work reported in this paper.
